# Enhanced Amphiphilic Profile of a Short β-Stranded Peptide Improves Its Antimicrobial Activity

**DOI:** 10.1371/journal.pone.0116379

**Published:** 2015-01-24

**Authors:** Giorgia Manzo, Mariano A. Scorciapino, Parvesh Wadhwani, Jochen Bürck, Nicola Pietro Montaldo, Manuela Pintus, Roberta Sanna, Mariano Casu, Andrea Giuliani, Giovanna Pirri, Vincenzo Luca, Anne S. Ulrich, Andrea C. Rinaldi

**Affiliations:** 1 Department of Chemical and Geological Sciences, University of Cagliari, Cittadella Universitaria, I-09042 Monserrato (CA), Italy; 2 Institute of Biological Interfaces (IBG-2), Karlsruhe Institute of Technology (KIT), POB 3640, 76021 Karlsruhe, Germany; 3 Department of Biomedical Sciences, University of Cagliari, Cittadella Universitaria, I-09042 Monserrato (CA), Italy; 4 Research & Development Unit, Spider Biotech S.r.l., I-10010 Colleretto Giacosa (TO), Italy; 5 Dipartimento di Scienze Biochimiche, “A. Rossi Fanelli”, Istituto Pasteur-Fondazione Cenci Bolognetti, Sapienza Università di Roma, Rome, Italy; 6 Institute of Organic Chemistry, Karlsruhe Institute of Technology (KIT), Fritz-Haber-Weg 6, 76131 Karlsruhe, Germany; University of Cambridge, UNITED KINGDOM

## Abstract

SB056 is a novel semi-synthetic antimicrobial peptide with a dimeric dendrimer scaffold. Active against both Gram-negative and -positive bacteria, its mechanism has been attributed to a disruption of bacterial membranes. The branched peptide was shown to assume a β-stranded conformation in a lipidic environment. Here, we report on a rational modification of the original, empirically derived linear peptide sequence [WKKIRVRLSA-NH_2_, SB056-lin]. We interchanged the first two residues [KWKIRVRLSA-NH_2_, β-SB056-lin] to enhance the amphipathic profile, in the hope that a more regular β-strand would lead to a better antimicrobial performance. MIC values confirmed that an enhanced amphiphilic profile indeed significantly increases activity against both Gram-positive and -negative strains. The membrane binding affinity of both peptides, measured by tryptophan fluorescence, increased with an increasing ratio of negatively charged/zwitterionic lipids. Remarkably, β-SB056-lin showed considerable binding even to purely zwitterionic membranes, unlike the original sequence, indicating that besides electrostatic attraction also the amphipathicity of the peptide structure plays a fundamental role in binding, by stabilizing the bound state. Synchrotron radiation circular dichroism and solid-state ^19^F-NMR were used to characterize and compare the conformation and mobility of the membrane bound peptides. Both SB056-lin and β-SB056-lin adopt a β-stranded conformation upon binding POPC vesicles, but the former maintains an intrinsic structural disorder that also affects its aggregation tendency. Upon introducing some anionic POPG into the POPC matrix, the sequence-optimized β-SB056-lin forms well-ordered β-strands once electro-neutrality is approached, and it aggregates into more extended β-sheets as the concentration of anionic lipids in the bilayer is raised. The enhanced antimicrobial activity of the analogue correlates with the formation of these extended β-sheets, which also leads to a dramatic alteration of membrane integrity as shown by ^31^P-NMR. These findings are generally relevant for the design and optimization of other membrane-active antimicrobial peptides that can fold into amphipathic β-strands.

## Introduction

Nowadays, many important pathogens have developed multi-drug resistance and are able to evade treatment with multiple antimicrobial classes, covering most, sometimes all, clinically usable antibiotics [1], [2]. The higher mortality rates and the increased lengths and costs of hospitalization are calling for the urgent development of new antibiotics [3]. Over the last two decades, much interest has focused on natural compounds known as antimicrobial peptides (AMP) or host defence peptides. This is a wide group of molecules expressed by multicellular organisms as effectors of the innate immune system. They are characterized by a wide spectrum of antimicrobial activity, ranging from Gram-positive to Gram-negative bacteria, from fungi to enveloped viruses and protozoa [4–6]. Despite a large variety in primary sequences and secondary structures, AMPs generally share a cationic character and their length usually does not exceed 50 residues. With a large proportion of hydrophobic amino acids, many of them also tend to assume globally amphipathic folds with clearly distinguishable hydrophilic and hydrophobic faces. While conventional antibiotics interact with specific bacterial targets (e.g., enzymes), allowing the pathogens to develop resistance relatively easily, AMPs usually act by physically destroying or permeabilizing the microbial plasma membrane through interactions with the lipids. This makes AMPs and their derivatives particularly suitable as novel antimicrobial drugs, because target substitution/modification, and thus resistance, is less likely to occur [7], [8].

However, despite intense research on AMPs, only a handful of peptides have been clinically approved yet. There are multiple hurdles that limit the direct development of a naturally occurring AMP into an applicable antibiotic. These include high manufacturing costs, susceptibility to protease degradation, and a reduced activity in the presence of salts at physiological concentrations [3], [9], [10]. Given the inherent limitations of using naturally occurring AMPs, two general approaches have emerged to overcome these obstacles, namely the modification of existing peptide sequences to make them proteolytically more stable, and the *de novo* synthesis of peptides and/or the design of synthetic molecules mimicking the properties and activities of natural AMPs (see [11] and references therein). Especially dendrimeric peptides have received much attention [12], [13] as branched macromolecules consisting of a core and a certain number of covalently attached functional units [14]. Usually, dendrimeric peptides display increased activity compared to their monomeric counterparts, probably because of the higher local concentration of the bioactive units [15]. Moreover, they have a greater stability towards proteases due to steric hindrance, thus increasing the peptides’ pharmacokinetic properties [16].

**Figure 1:**
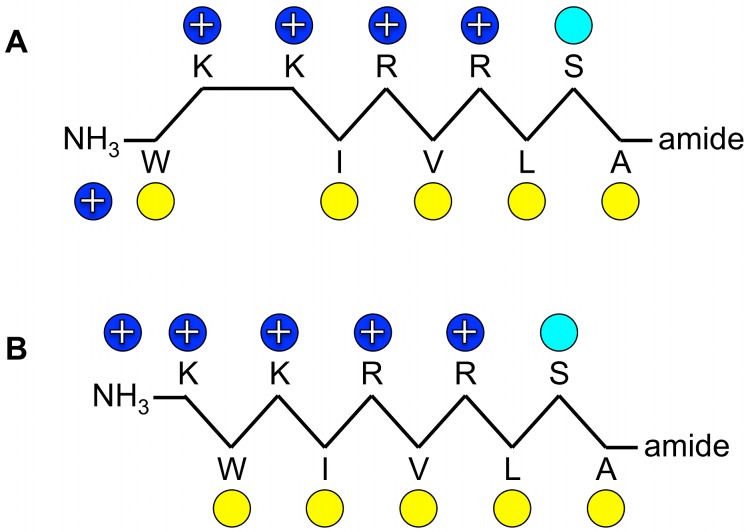
Schematic representation of the peptides. **(A)** Original SB056-lin, and **(B)** sequence-optimized β-SB056-lin. Yellow circles indicate hydrophobic residues, blue ones positively charged amino acids, and cyan indicates the polar serine residue.

Starting from a linear AMP sequence, originally identified by selecting a random phage library against *Escherichia coli* cells, a rational modification and optimization process led to the tetra-branched peptide known as SB041 [17]. This compound was found to be especially active against Gram-negative strains, and able to strongly bind *E. coli* and *Pseudomonas aeruginosa* lipopolysaccharide (LPS) *in vitro*. Further modifications of the primary sequence led to SB056, a novel AMP with a dimeric dendrimer scaffold. SB056 is highly active against Gram-negative bacteria, with a potency comparable to that of Colistin and Polymyxin B, but it shows a broader spectrum of activity, with a certain activity also against Gram-positive bacteria [18]. A biophysical characterization by circular dichroism (CD), nuclear magnetic resonance (NMR) and molecular dynamics (MD) simulations, combined with membrane affinity assays by lipid monolayer surface pressure experiments, revealed that this interesting peptide was indeed membrane-active by folding into a β-type conformation in lipidic environments [18]. The primary sequence [WKKIRVRLSA] of the peptidic part of the dendrimeric SB056 reveals a striking pattern of alternating hydrophilic and hydrophobic amino acids, with the exception of the first two residues that shall now be manipulated here (Fig. 1A). This sequence is largely compatible with an extended β-strand conformation on the membrane surface, such that the hydrophobic residues are embedded in the membrane and the hydrophilic residues are directed towards the lipid-water interface.

From such structural point of view, SB056 is reminiscent of the model peptide [KIGAKI]_3_ that had been specifically designed as an amphiphilic β-strand [19]. This regular peptide sequence exhibits a high antimicrobial activity, and it has also been suggested as a model system for studying membrane-induced amyloid formation. Recently, using electron microscopy and CD analysis, it has been shown to assemble as cross-β-sheet amyloid-like fibrils, both in solution and in the membrane-bound state [20], [21]. Solid-state ^19^F-NMR showed that it forms immobilized β-stranded aggregates on the lipid bilayer surface. The antimicrobial activity of the KIGAKI-type peptides thus seems to be related to the formation of amphipathic β-stranded aggregates on the bilayer surface, which in turn was found to be strictly dependent on the peptide length [22]. A series of peptides composed of KIGAKI repeats with lengths varying between 6 and 30 residues showed that the β-content of the bound molecules increased sigmoidally with peptide length, with a midpoint around 10–12 amino acids [22]. Peptides longer than 10 amino acids readily form β-structures, while much less β-conformation is observed in the shorter ones.

The peptidic part of the SB056 dendrimer contains only 10 residues and represents the lower limit for β-strand formation upon membrane binding. In the present work we have rationally optimized the profile of the linear SB056 sequence (SB065-lin) by interchanging the first two residues to obtain the perfectly regular amphipathic β-SB056-lin [KWKIRVRLSA-NH_2_] (Fig. 1B). Before investigating any effects in the context of the dimeric dendrimer scaffold, here we have focused on a comparison of the two linear analogues (SB056-lin and β-SB056-lin) in order to find out whether the strategy of improving the amphiphilic profile would really enhance the formation of β-stranded structures in lipid bilayers, and whether this feature could be used to improve also the antimicrobial activity. Various complementary biophysical techniques are applied to systematically characterize the two SB056 linear analogues.

## Materials and Methods

### Peptide synthesis

SB056-lin [WKKIRVRLSA-NH_2_] and β-SB056-lin [KWKIRVRLSA-NH_2_] were synthesized on a rink amide resin with an amidated C-terminus. A standard solid-phase peptide Fmoc/OtBu (9-fluorenylmethyoxy-carbonyl) strategy was employed as reported [18]. The crude peptides were checked by analytical RP-HPLC on a Jupiter Proteo analytical C12 column (4.6×250 mm) supplied by Phenomenex (Torrance, CA, USA), using 0.1% trifluoroacetic acid (TFA)/H_2_O as solvent A and 0.1% TFA/MeCN as solvent B, the concentration of the latter going from 5% to 95% V/V over 14 min at a flow rate of 1.0 mL/min. Peptides were purified on a Jupiter Proteo semi-preparative C12 column (10×250 mm) as the major peak. MALDI-TOF mass spectrometry (Bruker Daltonik, Bremen, Germany) was performed using sinapinic acid. For solid-state ^19^F-NMR analysis, the reporter group *L*-3-(trifluoromethyl)-bicyclopent-[1.1.1]-1-ylglycine (CF_3_-Bpg) was substituted into the position of Val6 [23], [24]. For CD and solid-state NMR investigations, these peptides were purified on a Vydac C18 preparative column using a fluorine-free solvent mixture composed of water/acetonitrile, supplemented with 5 mM HCl as previously described [20], [25].

### Minimum Inhibitory Concentration determination

The minimum inhibitory concentration (MIC) of SB056-lin and β-SB056-lin was determined against two Gram-negative and two Gram-positive reference bacterial strains: *E. coli* ATCC 25922, *P. aeruginosa* ATCC 27853, *Staphylococcus aureus* ATCC 25923 and *Enterococcus faecalis* ATCC 29212, using a modified two-fold broth dilution assay (AlamarBlue assay) [26]. Bacteria were grown in Mueller-Hinton (MH) broth at 37°C with continuous shaking at 200 rpm, up to an optical density at 550 nm of 2.0. Then, the culture was diluted with MH broth up to a final bacterial concentration of 10^6^ CFU/mL. MIC values were determined in a sterile 96-well polystyrene microtiter plate (total volume 100 μL), where the peptide was added to each well by following a serial dilution of the stock solution (in ethanol/MilliQ water 1/1 v/v). Finally, 50 μL of bacterial suspension were inoculated into each well (to a final bacterial concentration of 5·10^5^ CFU/mL). The plates were incubated for 22 h at 37°C, and bacterial growth was observed with the AlamarBlue assay [26]. The MIC value (μg/mL) corresponds to the peptide concentration at which no bacterial growth was observed.

### Hemolytic activity

The hemolytic activity of the peptides was determined using fresh human erythrocytes from healthy donors. Blood was centrifuged and the erythrocytes were washed three times with 0.9% NaCl. Peptides dissolved in water were added to the erythrocyte suspension (5%, v/v), at a final concentration ranging from 0.25 to 64 μg/mL in a final volume of 100 μl. Samples were incubated with agitation at 37°C for 40 min. The release of hemoglobin was monitored by measuring the absorbance (Abs) of the supernatant at 415 nm. Control for zero hemolysis (blank) consisted of erythrocytes suspended in 0.9% NaCl. Hypotonically lysed erythrocytes (in water) were used as a standard for 100% hemolysis. The percentage of hemolysis was calculated using the following equation: % hemolysis = [(Abs sample–Abs blank)/(Abs total lysis–Abs blank)] × 100. The results are the mean of three independent experiments.

### Preparation of lipid vesicles

The lipids POPC (1-palmitoyl-2-oleoyl-*sn*-glycero-3-phosphocholine) and POPG (1-palmitoyl-2-oleoyl-*sn*-glycero-3-phospho-(1′-*rac*-glycerol), sodium salt) were purchased from Avanti Polar Lipids (Alabaster, AL, USA) and used without further purification. Large unilamellar vesicles (LUV) and small unilamellar vesicles (SUV) were used for fluorescence spectroscopy and CD analysis, respectively. In order to vary the negative surface charge of the liposomes, we employed mixtures of the zwitterionic POPC and the anionic POPG at different molar ratios, namely 0% POPG, 25% POPG, 50% POPG, 75% POPG, and 100% POPG. Weighed amounts of POPC and POPG were dissolved in chloroform/methanol (1/1 v/v). The solvent was evaporated under a gentle stream of nitrogen, followed by vacuum overnight. The resulting lipid film was hydrated with 10 mM phosphate buffer (PB, pH 7.4) and multilamellar vesicles (MLV) were formed by vortexing 5× 1 min, followed by 5 freeze-thaw cycles. Afterwards, LUVs were prepared by extrusion, by passing the MLV dispersion 11× through two polycarbonate filters (Whatman, Avanti polar lipids, Inc.) with pore size of 400 nm and then 100 nm, using an Avanti mini-extruder (Avanti polar lipids, Inc.). SUVs were obtained by sonication of the MLV dispersion 4× 4 min in a high-power ultrasonic bath with a beaker-shaped sonotrode (UTR 200, Hielscher, Germany). The water in the ultrasonic bath was cooled down to room temperature (with ice) between the sonication steps to avoid overheating.

### Fluorescence spectroscopy

Lipid binding assays were performed by fluorescence spectroscopy with an LS55 Luminescence Spectrometer (Perkin-Elmer, Waltham, MA, USA) equipped with a thermostated cuvette holder. LUVs with different POPC/POPG molar ratios were prepared as described above. Peptide was added at a final concentration of 1 μM to the buffered liposomal dispersion, and aliquots of the liposome stock solution were diluted to obtain the following lipid/peptide molar ratios: 0, 10, 15, 20, 30, 40, 60, 80, 100, 150, 200, 400. The intrinsic tryptophan fluorescence was measured at 27°C (i.e. well above lipid phase transition temperature of −2°C) by recording the emission spectrum between 300 and 450 nm. The excitation wavelength was set to 280 nm, and beam entry and exit slit width was set to 5 mm. The LUV dispersion before peptide addition yielded the reference spectrum for background subtraction.

### Circular dichroism spectroscopy

Synchrotron radiation (SR) CD was used to investigate the secondary structure of the peptides, because the strong background absorption of the unsaturated lipids and the unfavorable scattering of the SUVs would have lead to poor spectral quality if conventional CD had been used. SRCD spectra were collected at the UV-CD12 beamline of the ANKA storage ring (KIT, Germany). The beamline components and its experimental end-station have been described in detail [27]. Following the closure of SRS (Daresbury Laboratory, UK) in 2008, the beamline that had been formerly known under the name of “CD12”, was transferred and adapted to ANKA. Weighed amounts of peptide were dissolved in 10 mM PB (pH 7.0) to obtain stock solutions at a concentration of 25 mg/mL. SUVs at different POPC/POPG ratios were prepared as described above. SRCD samples were prepared by adding a proper aliquot of peptide stock solution to the liposome dispersion to obtain a final lipid/peptide molar ratio of 25 (~1.6 mM peptide, ~40 mM lipids). Measurements were performed on a 3 μL aliquot of the sample in a “Birkbeck-type” demountable CaF_2_ cell [28] with a path length of 13.1 μm (HELLMA, Müllheim, Germany), acquiring the spectrum between 260 and 180 nm at 0.5 nm intervals. Spectra were recorded at 20°C, i.e. well above lipid phase transition temperature (−2°C), using a cell holder thermostated by Peltier elements. A scan rate of 14 nm/min, 1 nm spectral bandwidth, 0.3 s lock-in time constant, and 1.5 s dwell time were applied. Three spectra were acquired and averaged for each sample. The average spectrum was corrected by subtracting the corresponding spectrum of SUV dispersion in the absence of peptide.

Conventional CD spectroscopy was also employed for the sake of comparison. Spectra were recorded on a J-815 spectropolarimeter (JASCO, Groß-Umstadt, Germany). Similarly to the SRCD experiments, peptide samples were prepared by adding a proper aliquot of a peptide stock solution to the liposome dispersion to obtain a final lipid/peptide molar ratio of 25 (~40 μM peptide, ~1.0 mM lipids). Measurements were performed in quartz glass cells (Suprasil, Hellma) of 1 mm path length between 260 and 180 nm at 0.1 nm intervals. Spectra were recorded at 20°C using a water-thermostated rectangular cell holder. Three repeat scans at a scan-rate of 10 nm min^−1^, 8 s response time and 1 nm bandwidth were averaged for each sample and for the baseline of the corresponding peptide-free sample. After subtracting the baseline from the sample spectra, CD data were processed with the adaptive smoothing method, which is part of the Jasco Spectra Analysis software.

### Solid-state NMR analysis

All solid-state NMR measurements of the CF_3_-Bpg labeled peptides were performed on a Bruker Avance 500 MHz spectrometer (Bruker Biospin, Karlsruhe, Germany). Macroscopically oriented samples were prepared as described previously [20], [29]. Peptides (0.2 to 0.5 mg) were dissolved in methanol/water, while the lipids were solubilized in chloroform/methanol (1:1). Different amounts of lipid were employed to obtain the desired lipid-to-peptide ratio, by adding the clear lipid solution to the clear peptide solution. The resulting solution was vortexed, sonicated for 1 min, and uniformly spread over 10–25 thin glass plates (9 mm × 7.5 mm). The plates were dried under air, and residual solvent was removed under vacuum overnight. The glass plates with the dry peptide-lipid films were stacked and hydrated overnight at 48°C in a humid chamber using saturated K_2_SO_4_ solution (96% humidity). The hydrated stack was wrapped in parafilm and thin polyethylene film to avoid drying of the sample. All experiments were performed at 308 K. Solid state ^19^F-NMR experiments were performed with a flat-coil ^19^F/^1^H probe, using an anti-ringing sequence with a 90° pulse of 2.5 μs, and with a relaxation delay time of 1s, 500 kHz spectral width, and proton decoupling using ttpm20 [30]. Usually 7000 to 50000 scans were recorded, and each spectrum was referenced to a 100 mM NaF solution for which the ^19^F-NMR signal was set to −119.5 ppm. ^31^P-NMR was used to check the quality of the phospholipid orientation in the samples, using a Hahn echo sequence with phase cycling. Typically, 128 to 512 scans were recorded with a relaxation delay time of 2 s, and with a spectral width of 100 kHz. Protons were decoupled using ttpm20.

## Results and Discussion

### Antimicrobial activity and hemolysis

Table 1 shows the MIC values determined for both peptides (SB056-lin and β-SB056-lin) against four standard bacteria strains, the Gram-negative *E. coli* and *P. aeruginosa*, and the Gram-positive *S. aureus* and *E. faecalis*. Values from the literature [18] for Polymyxin B and Colistin are reported for comparison. In the present work, the original SB056-lin sequence [WKKIRVRLSA-NH_2_] has been optimized by inverting the first two residues to give a perfectly regular amphiphilic sequence [KWKIRVRLSA-NH_2_]. This strategy was based on the expectation that better membrane binding and a more favorable β-strand assembly would lead to enhanced disruption of the lipid bilayer, as has been reported recently for various AMPs in a vesicle fusion assay combined with CD analysis [31]. The MIC values clearly show that the sequence optimization significantly increased the antimicrobial activity against both Gram-positive and Gram-negative bacteria. The simple amino acid interchange lead to a 3–4 fold increase in activity of β-SB056-lin, bringing it close to that of Polymyxin B and Colistin which are conventionally used against Gram-negative strains. While the two conventional antibiotics are essentially inactive against Gram-positive bacteria, β-SB056-lin shows a very broad spectrum of activity, being able to inhibit their growth almost as well as in the case of Gram-negative bacteria.

**Table 1:** MIC values of SB056-lin and β-SB056-lin against two Gram-negative and two Gram-positive bacterial strains.

	**Microorganism/Strain**			
	***E. coli***	***P. aeruginosa***	***S. aureus***	***E. faecalis***
**MIC (μg/mL)**	**ATCC 25922**	**ATCC 27853**	**ATCC 25923**	**ATCC 29212**
SB056-lin	64	64	64	256
β-SB056-lin	4	16	8	64
^a^ Polymyxin B	0.25–2	0.5–2	128	>128
^a^ Colistin	0.25–2	0.5–2	>128	>128

Values from the literature for Polymyxin B and Colistin are presented for comparison [18].

^a^ Ranges obtained against different strains.

Table 2 reports the measured lytic activity of SB056-lin and β-SB056-lin against human red blood cells. While the hemolytic activity of the original SB056-lin is very low at any tested concentration (max 4% at 64 μg/mL), sequence regularization led to a slight increase of cytotoxicity for β-SB056-lin at high concentrations (max 11% at 64 μg/mL), while negligible hemolytic activity was maintained at peptide concentrations close to the MIC values for both *E. coli* and *S. aureus* (Table 1). These findings are in line with the results of the membrane binding affinity assay, that clearly demonstrated an enhanced affinity of β-SB056-lin also for zwitterionic lipid bilayers (see below).

**Table 2:** Hemolytic activities of SB056-lin and β-SB056-lin.

**% Hemolysis**									
**Peptide**	**Peptide concentration (μg/mL)**								
	**64**	**32**	**16**	**8**	**4**	**2**	**1**	**0.5**	**0.25**
SB056-lin	4.1 ± 1.2	2.1 ± 0.1	2.0 ± 1.6	2.0 ± 1.4	2.0 ± 1.1	0.0 ± 0.0	ND	ND	ND
β-SB056-lin	11.1 ± 0.9	10.9 ± 1.9	6.4 ± 1.6	5.9 ± 0.2	2.4 ± 1.1	2.1 ± 0.5	2.0 ± 1.2	ND	ND

ND = Not determined. Values are means of three independent measurements, ± SD.

### Peptide binding examined by fluorescence spectroscopy

Tryptophan fluorescence is readily used to monitor the binding of peptides to lipid model membranes, as the tryptophan side chain tends to enter a more hydrophobic environment. Typically, this leads to a blue shift and an increase in the quantum yield of fluorescence [32], [33]. We have investigated the two SB056 analogues in the presence of LUVs prepared with different molar ratios of zwitterionic/anionic lipids, given that bacterial membranes are characterized by a much higher content of anionic lipids than eukaryotic ones [4], [34]. Furthermore, the content of anionic lipids in Gram-negative bacteria is around 30%, while it is 70% or more in Gram-positive ones [35], [36]. POPC was chosen as the uncharged phospholipid, and the corresponding phosphoglycerol (POPG) was employed as the anionic one.

The fluorescence intensity is known to depend on many different factors that are hard to take into account quantitatively. For instance, upon binding the tryptophan residues could suffer from fluorescence self-quenching if peptide oligomerization occurs. Another source of quenching might be the interaction between tryptophan and cationic groups on the peptide [37], like the tryptophan-flanking lysines in the peptides under investigation. Moreover, quenching could also be due to the charged head group of POPG interacting with the tryptophan π-orbitals [32]. All these contributions depend on the specific secondary structure adopted by the peptide, on its oligomerization state, and on its alignment in the bilayer. In the present case, accurate structural information about the vesicle-bound state is not available, the position of tryptophan differs for the two peptides, and the POPG content of the lipid vesicles was systematically varied. Considering all these factors, even if the fluorescence intensity as a function of the lipid/peptide ratio ([L]/[P]) could in principle be related to the peptide binding constant [33], no attempt was made to estimate the latter. However, the tryptophan blue shift can still provide important qualitative information about the relative binding strength/affinity of different peptides for the same membrane model. Similarly, the relative binding affinities of a peptide for differently charged membranes can be evaluated. The tryptophan emission wavelength (λ) usually decreases with increasing [L]/[P] until saturation is reached [32], [33]. Although the maximum difference (Δλ_max_) between saturation and starting λ (i.e. the wavelength in the absence of lipids) depends on several factors (similarly to fluorescence intensity), the higher the peptide binding affinity, the lower the saturation [L]/[P].

**Figure 2:**
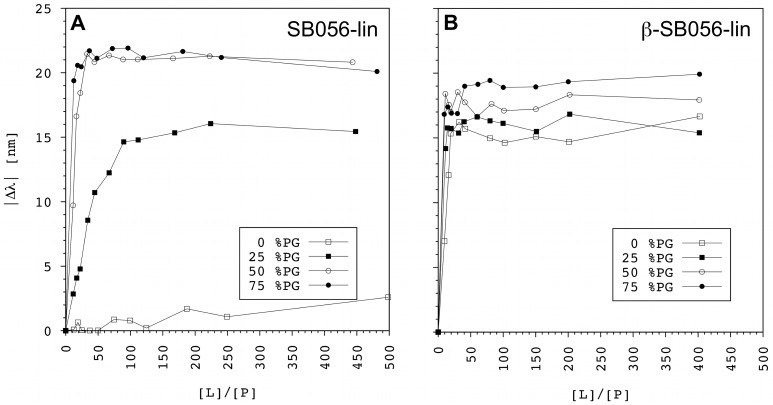
Membrane binding curves. The blue shift of tryptophan fluorescence emission |Δλ| is plotted as a function of [L]/[P] for **(A)** SB056-lin, and **(B)** β-SB056-lin, in the presence of differently charged POPC/POPG LUVs. The lower the [L]/[P] needed to reach saturation, the higher is the peptide binding affinity.

Fig. 2 shows the results obtained for the two systems investigated here. The absolute value of Δλ is plotted as a function of the [L]/[P] molar ratio. As expected, the binding affinity increases with increasing POPG content in the vesicles, indicating that the interaction of the cationic peptides with the membrane increases with the anionic character of the latter. Comparison of the two SB056 analogues shows interesting differences, even though the two peptides bear exactly the same net charge (+5). SB056-lin has a marked binding affinity only for the two highest POPG contents investigated (i.e. 50% and 75%), whereas a comparable tryptophan fluorescence blue-shift was observed already at 25% POPG for the optimized β-SB056-lin. Even more surprisingly, β-SB056-lin shows considerable binding even to pure POPC liposomes, whereas SB056-lin has essentially no affinity for this uncharged membrane.

These results suggest that, even though electrostatics play a major role, also the folding and aggregation preferences of the particular sequence can significantly affect the binding properties. Electrostatic interactions are usually required to bring the peptide from the aqueous environment close to the membrane. However, peptide binding (i.e. the equilibrium between the bound- and unbound-states) will be enhanced by a favorable amphiphilic profile and by peptide-peptide interactions, such as β-sheet aggregation in the bound state. In our case, the alternating pattern of charged/polar and hydrophobic residues along the sequence of β-SB056-lin conveys an ideal amphiphilic character when bound as an extended β-strand, which can explain its high affinity even for pure POPC.

### Circular dichroism spectroscopy—peptide secondary structure

**Figure 3:**
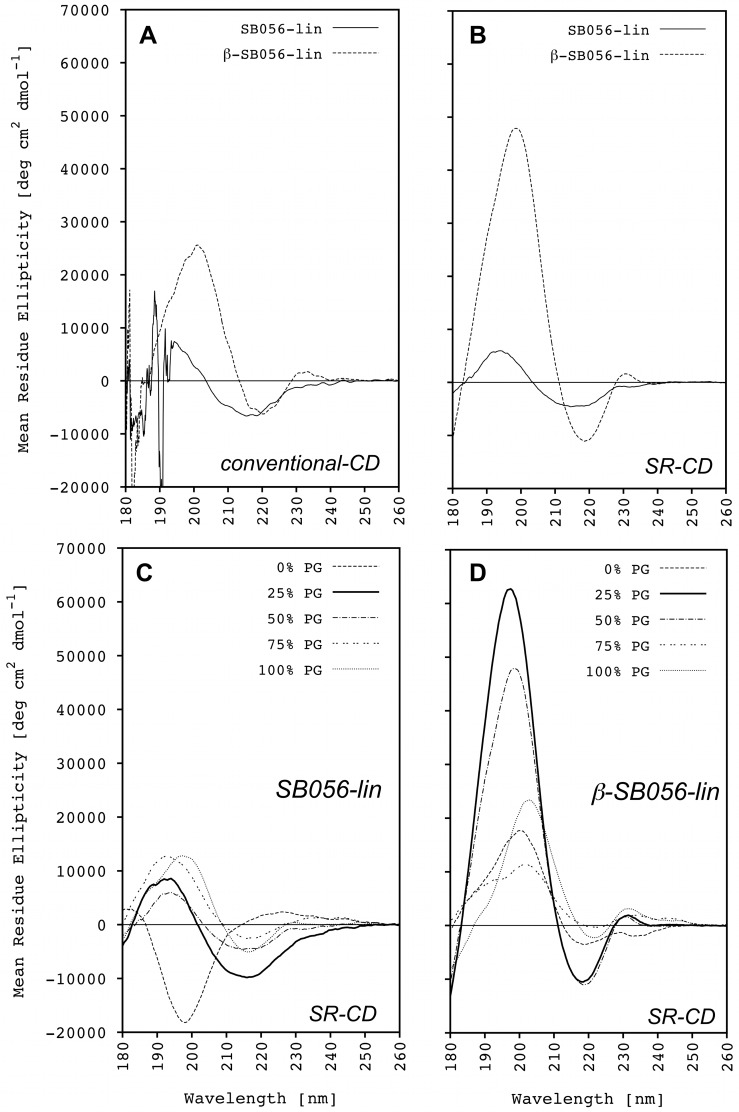
Circular Dichroism analysis. **(A)** Conventional CD spectra of both SB056-lin and β-SB056-lin in the presence of POPC/POPG (1/1 mol/mol) SUVs. **(B)** SRCD spectra of both SB056-lin and β-SB056-lin in the presence of POPC/POPG (1/1 mol/mol) SUVs. SRCD spectra of **(C)** SB056-lin and **(D)** β-SB056-lin in the presence of differently charged SUVs.

The secondary structure adopted by the two peptides in the presence of differently charged POPC/POPG vesicles was investigated by CD spectroscopy. Fig. 3A, for instance, shows the conventional CD lineshapes obtained for SB056-lin and β-SB056-lin in the presence of POPC/POPG (1/1 mol/mol SUVs). The positive band at ~200 nm and the negative band at ~220 nm are visible and indicate a β-type conformation for both peptides. As expected, the spectrum of the optimized β-SB056-lin is much more intense, indicating a more characteristic and regular β-strand conformation than for the original SB056-lin analogue. However, this example clearly suffers from severe spectral distortions that are commonly encountered on conventional CD instruments when measuring peptide/lipid vesicle samples at wavelengths <200 nm, which can make the resulting lineshapes very difficult to interpret and possibly even render the technique useless in some cases. These distortions are mostly due to an insufficient intensity of the incident radiation below 190 nm, and due to severe scattering artifacts even when small unilamellar liposomes are employed (see CD section in Materials and Methods).

Synchrotron radiation CD (SRCD) is based on the same basic principle as conventional CD, but it offers numerous advantages that have been described in detail [38–42]. At wavelengths below 200 nm the photon flux in SRCD is orders of magnitude more intense than what is typically available in conventional instruments, allowing spectra to be obtained well into the vacuum ultraviolet region. Since additional electronic transitions occur in that wavelength region, these data also contain more information than conventional CD spectra. In addition, the greater intensity of the synchrotron light source provides a better signal-to-noise ratio (S/N) in the spectra collected with SRCD instruments. Fig. 3B, for instance, shows two spectra recorded at the UV-CD12 beamline of the ANKA synchrotron, to be compared to those in Fig. 3A obtained with conventional CD instrumentation. The significant improvement in S/N achieved with SRCD is evident, as these spectra are free from distortions below 190 nm.

Fig. 3C and 3D show the SRCD spectra obtained for SB056-lin and β-SB056-lin, respectively, in the presence of differently charged POPC/POPG vesicles. The data show that both peptides have a clear tendency to assume a β-type conformation in the presence of lipid vesicles. This is in agreement with a previous report on the folding propensity of SB056 peptides [18]. The characteristic positive band at 195–200 nm and the negative one at 215–220 nm are clearly distinguishable in most of the spectra. However, a relatively low intensity and deviations from a canonical β-sheet spectrum are sometimes observed, which can be attributed to peptide-peptide interactions and absorption flattening (see below). These data suggest that (i) structural order and/or peptide aggregation depends on the negative charge in the bilayer, (ii) the two SB056 analogues respond differently to a variation in membrane charge, and (iii) they fold/aggregate in different ways.

In contrast to all other spectra, the spectrum of SB056-lin in pure POPC vesicles (Fig. 3C) shows an intense negative band at ~198 nm and weak positive ellipticities around 225 nm. The negative band can be attributed to a random coil conformation, even though both spectral features are also similar to those characteristic of a polyproline II (PPII) secondary structure [38]. A small blue-shift of the negative band from ~200 nm, an increase in the negative mean residue ellipticity, which is typically observed in PPII peptides, and a small but pronounced positive band around 225 nm, usually serve as criteria to distinguish between random coil and PPII conformation in conventional CD spectra. Here, synchrotron radiation allows us to obtain reliable CD spectra below 190 nm and thereby adds another feature to discriminate both conformations. While PPII peptides are usually characterized by negative ellipticities in the range of 180–190 nm, a random coil conformation is distinguished by small positive ellipticities around 180 nm [38]. Only by using SRCD, we were able to confirm that SB056-lin is predominantly unstructured as a random coil in the presence of pure POPC vesicles (Fig. 3C). This finding is fully compatible with the negligible binding observed with fluorescence spectroscopy.

Although peptide binding was found to increase with increasing the negative charge in the bilayer, CD suggests that folding of SB056-lin is poor in all cases investigated here, up to 100% POPG content in the vesicles (Fig. 3C). The spectral lineshape indicates some kind of β-type structure, with a positive band at ~195 nm and a negative one at ~217 nm, but the relatively low amplitude of these bands suggests that the formation of regular β-strands on the bilayer can be ruled out. It appears plausible that increasing the negative charge of the vesicle favors binding, such that more and more SB056-lin monomers accumulate on the vesicle surface. However, they seem to maintain an intrinsic conformational disorder due to their non-optimized sequence.

In contrast, the sequence-optimized β-SB056-lin peptide exhibits a strong binding affinity even for pure POPC vesicles. Accordingly, its corresponding SRCD spectrum (Fig. 3D) is clearly not indicative of a random coil structure. Also in this case, a β-type conformation can be inferred, though regular β-sheets can be excluded. A dramatic difference is also observed compared to the non-optimized analogue once the negative charge on the vesicles is being increased. The SRCD spectrum obtained in the presence of 25% POPG (Fig. 3D) is very intense and clearly indicates that the peptide folds into well-ordered β-strands. When the whole system approaches electro-neutrality, which corresponds to 25% POPG, the maximum spectral intensity is observed.

When the negative charge of the vesicles is increased further, thus moving to an excess of anionic charges in the whole peptide-lipid mixture, deviations from the canonical β-sheet spectral lineshape are again observed (Fig. 3D). Decreasing intensity and broadening of both the positive and the negative CD bands leads to a shift in the x-axis intersection point towards higher wavelengths, and both bands also exhibit a slight red shift. These changes can be attributed to absorption flattening and differential scattering phenomena. Absorption flattening is a consequence of the non-random distribution of chromophores within the sample, i.e. to differential absorption from different portions of the sample. In peptide-membrane samples it occurs when the peptide chromophores are sequestered in discrete regions of the sample with high local density. The extent of absorption flattening is proportional to the concentration of absorbers in each particle [43]. Thus, with increasing binding, more and more peptides seem to form extended β-pleated aggregates on the bilayer and thereby enhance the absorption flattening effects in the CD spectrum. Differential scattering of the incident radiation becomes more and more important with increasing aggregate size [44]. Thus, the more extended the β-sheets, the more severe is the loss in spectral intensity.

The present CD/SRCD data clearly show a different behavior for the two SB056 analogues. The non-optimized SB056-lin is electrostatically attracted and binds in not so well-ordered β-strands. The optimized β-SB056-lin, on the other hand, forms regular β-strands that assemble into extended β-sheets, whose size increases with the negative charge of the bilayer.

### Solid-State NMR analysis

Solid-state NMR is particularly well suited to study peptide-lipid interactions, as demonstrated for various α-helical peptides, for unstructured membrane-bound peptides, and for monomeric β-strands and oligomeric β-sheets [20], [21], [29], [45–47]. Theoretical details about solid-state NMR have been described previously in detail ([48–50] and references therein). In particular, the high sensitivity of ^19^F-NMR allows us to readily distinguish binding, folding, aggregation, and transitions into amyloid-like fibrils in the presence of membranes. It has been previously demonstrated that peptide structure and function remain unperturbed when a single hydrophobic residue in a membrane active peptide is selectively replaced with (CF_3_-Bpg or CF_3_-Phg) [51–53]. Indeed, also in the present case the ^19^F-labeled [WKKIR(CF_3_-Bpg)RLSA] and [KWKIR(CF_3_-Bpg)RLSA] exhibit MIC values similar to those of the unlabeled peptides (data not shown).

^19^F-NMR analysis relies on the direct read-out of the dipolar splitting of the ^19^F-labeled reporter group on the peptide, which reveals the alignment and mobility of the CF_3_-Bpg side chain that is rigidly attached to the backbone, and which can therefore reflect the behaviour of the entire (folded) molecule. Especially in the case of β-stranded peptides, several characteristic scenarios can be readily distinguished based on a single ^19^F-labeled position [20], [21], [29]. For example, an isotropic signal is usually indicative of a highly mobile peptide in a disordered state that is not bound to the lipid bilayer at all. The largest possible splitting of +17 kHz, on the other hand, indicates that the CF_3_-Bpg side chain is inserted straight into the membrane and immobilized parallel to the lipid acyl chains. This is a signature of oligomeric β-strands that are assembled on the membrane surface in an amyloid-like manner. Any splitting in between, that is reduced by a factor of 2 upon tilting the sample by 90°, indicates that the peptide is free to undergo lateral diffusion in the bilayer and has not aggregated, thus reflecting a monomeric or lower oligomeric state. In contrast, a dipolar splitting of −7.5 kHz with a powder-like lineshape means that the peptide has aggregated without preferential orientation and is immobilized. At the same time, solid-state ^31^P-NMR can be used to assess the influence of the peptide on the phospholipid bilayer by observing any perturbance induced by the peptide. A higher antimicrobial activity of a β-stranded peptide may be expected to correlate with a greater perturbance of the membrane, as has been previously proposed based on a vesicle fusion assays [31].

**Figure 4:**
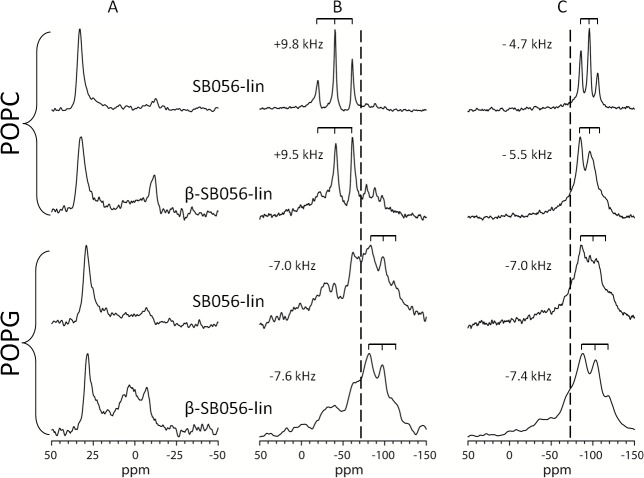
Solid-state NMR spectra of SB056-lin and β-SB056-lin. The peptides were labeled at positionVal-6 with CF_3_-Bpg, and solid-state NMR spectra were measured at 35°C in POPC or POPG at a [L]/[P] of 25. **(A)** ^31^P-NMR spectra of oriented samples at 0° tilt. **(B)** ^19^F-NMR spectra at 0° sample tilt. **(C)** ^19^F-NMR at 90° sample tilt. The isotropic position is marked with a dashed line, and the value of the dipolar splitting is indicated along with peptide names.

Our ^31^P-NMR results show that SB056-lin induces no significant bilayer perturbation in POPC at a [L]/[P] of 25. ^19^F-NMR exhibits a well-defined triplet that gets reduces by a factor of 2 upon tilting the sample, indicating that the peptide is mobile and most likely in a monomeric state (Fig. 4). Under the same conditions, β-SB056-lin is also mobile, but ^31^P-NMR shows considerable bilayer perturbance (Fig. 4). When anionic POPG is added to the POPC matrix, the binding-aggregation equilibrium becomes more complex, and the NMR spectra cannot be easily deconvoluted (data not shown). In pure POPG, finally, SB056-lin causes only slight perturbation, while β-SB056-lin induces massive disturbance in the lipid bilayer at a [L]/[P] of 25. ^19^F-NMR of both SB056-lin and β-SB056-lin shows powder spectra, suggesting that the peptides have aggregated (Fig. 4). This membrane perturbing effect can be directly correlated with the MIC values measured of the two peptides, given the presence of negatively charged lipids in bacterial membranes.

## Summary and Conclusions

Antimicrobial peptides in general, and dendrimeric analogues in particular, represent an attractive research avenue for developing novel antibiotics [54]. However, there are still many open questions concerning their mode of interaction with bacterial membranes [55], [56]. Starting from the dendrimeric antimicrobial peptide SB056, which had been originally obtained by semi-empirical optimization after high-throughput screening of a phage library [15], here we have regularized its amino acid sequence in order to enhance its amphipathic profile and, in turn, its antimicrobial activity. Inspection of the original sequence [WKKIRVRLSA] revealed that, with the only exception of the first two residues, it consists of alternating hydrophobic and cationic/polar amino acids. In this respect, it resembles the designer-made β-stranded model sequence [KIGAKI]_3_ [19] and several other peptides that are known to form β-sheets in the membrane-bound state, which have been characterized in detail [20], [22]. Recently, the lipid-induced propensity of SB056 to fold as an amphiphilic β-strand has also been demonstrated using a combined experimental and computational approach [18].

**Figure 5:**
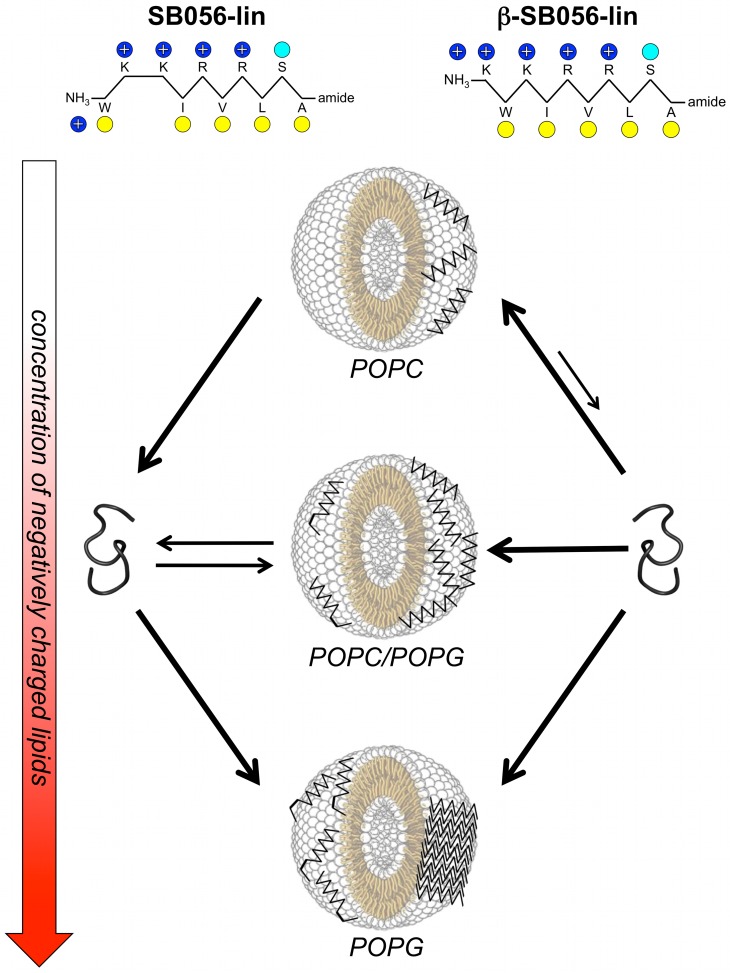
Summary of peptide-lipid interactions. The proportion of anionic lipids in the vesicles is increased from top to bottom. The behavior of the original SB056-lin peptide is represented on the left hand side, and the sequence-optimized β-SB056-lin on the right. Black arrows of different length and thickness are used to indicate the different binding equilibria. SB056-lin binds only to anionic bilayers, and in a not so well-ordered β-stranded conformation. The sequence optimized β-SB056-lin, on the other hand, forms regular β-strands that self-assemble into extended β-sheets when the negative charge of the bilayer exceeds electro-neutrality of the peptide-lipid system.

In the present work, we predicted that enhancement of the SB056 amphipathic profile would lead to favorable β-sheet aggregation on the lipid bilayer, and thereby to an enhanced antimicrobial activity. With the first two residues interchanged, β-SB056-lin [KWKIRVRLSA] bears the same net positive charge (+5) as the original linear peptide, but it maintains a perfectly alternating pattern of charged/polar and hydrophobic amino acids throughout the sequence (Fig. 1). Our microbiological assays showed an improved antimicrobial activity against both Gram-positive and Gram-negative bacteria, with a broader spectrum than other well-known clinically used peptidic antibiotics such as Polymyxin B and Colistin (Table 1). The predicted optimization was thus successful, leading in a straightforward manner to our second aim, i.e. to understand how such a relatively simple structural modification could affect the antimicrobial activity so prominently. Various complementary biophysical techniques were used to study the binding, folding, aggregation and mobility of the two SB056 analogues, when interacting with differently charged membrane models. These data provided evidence for direct structural reasons to explain the dramatically improved antimicrobial potency of the regularized sequence. Our findings are schematically summarized in Fig. 5.

The membrane binding affinity of both peptides was found to increase with increasing the anionic/zwitterionic lipid ratio in the vesicles, as expected for cationic peptides. However, the sequence-optimized β-SB056-lin showed a remarkable binding even to pure POPC bilayers, unlike the original SB056-lin. This observation indicates that, besides electrostatics, also the global amphipathicity of the folded peptide can be sufficient to stabilize the bound state, leading to dramatic differences in peptide behavior after interchanging just two residues out of ten. On the other hand, the greater affinity of the sequence-regularized analogue for zwitterionic membranes is also responsible for its greater cytotoxicity against human erythrocytes, an aspect that should be taken into consideration when any further development of these peptides into therapeutically valuable compounds will be attempted. When the proportion of anionic lipids in the target membrane is increased, more and more peptide monomers were found to bind. Both SB056-lin and β-SB056-lin adopt a β-stranded conformation, as expected. However, the former maintains a considerable degree of structural disorder, given that the position of the positive charges and the hydrophobic residue on the N-terminus cannot be accommodated in a straight amphipathic β-strand. Since the peptide is relatively short, an adaptation of the first two residues to a regular amphiphilic pattern has a profound effect on the overall peptide conformation and self-assembly in the membrane bound state.

When the peptide-lipid system approaches electro-neutrality (around 25% POPG in our case), the amphipathically-enhanced β-SB056-lin is seen to form very well-ordered β-strands. They self-assemble into more and more extended β-sheets when the proportion of anionic lipids is further increased in the sample. At the same time, the mobility of these extended peptide aggregates decreases, as shown by ^19^F-NMR. Thus, the enhanced antimicrobial activity of the regularized analogue appears to be correlated with the formation of such extended β-sheets on the bacterial membrane. Although elucidation of the detailed mode of action will still require further investigations, it is plausible that such highly positively charged peptides can induce anionic lipid clustering in the bilayer [57]. Therefore, a high local concentration of membrane-bound β-stranded peptides that can assemble into extended β-sheets seems to be the basis of the antimicrobial action of this class of peptides. The strong membrane perturbing effects that have been seen above by ^31^P-NMR and in earlier lipid fusion assays [31] can be attributed to this kind of self-assembly, with dramatic effects of membrane permeability, stability and/or depolarization.
